# Intraoperative Transesophageal Echocardiography to Monitor for Pulmonary Emboli in a Pediatric Patient Undergoing Undifferentiated Embryonal Sarcoma of the Liver Resection

**DOI:** 10.1155/2021/5532028

**Published:** 2021-06-19

**Authors:** Irim Salik, Nicolas Lamper, Bhupen Mehta, Kar-Mei Chan

**Affiliations:** Department of Pediatric Anesthesiology at Westchester Medical Center, New York Medical College, Valhalla, NY, USA

## Abstract

A minimally invasive monitoring technique, intraoperative transesophageal echocardiography (TEE), has been utilized to provide real-time data on volume status and ventricular function in patients undergoing liver transplantation. In this case, TEE was utilized in an 8-year-old female undergoing undifferentiated embryonal sarcoma of the liver resection to monitor for pulmonary emboli, particularly a saddle embolus. In addition to visualization of cardiac structures, TEE can also be utilized to monitor the liver, lungs, spleen, and kidneys. Monitoring for echocardiographic findings of pulmonary embolism in this high-risk patient was an integral part of effective intraoperative management.

## 1. Introduction

Undifferentiated embryonal sarcoma of the liver (UESL) is a rare tumor and the third most common hepatic malignancy in the pediatric population after hepatoblastoma and hepatocellular carcinoma, with an incidence of 1 case per million [1]. UESL commonly presents in children aged 6 to 10 years, as a solitary nodule in the right liver lobe with extrahepatic metastasis seen in 5–15% of patients to the lung, diaphragm, heart, and peritoneum. The malignancy is derived from mesenchymal tissue, with chromosomal abnormalities of 1q, 5p, 6q, 14, 9p, and 11p [52, 53] described in the literature [1]. Patients largely present with nonspecific symptoms including abdominal pain, nausea, fever, and anorexia. A worsened prognosis is associated with tumor rupture, local recurrence, and metastatic disease [2]. Patients with UESL have a 5-year survival rate over 70% with prompt partial hepatectomy and adjuvant chemotherapy [3].

Owing to the relative safety of TEE along with its utility in identifying cardiovascular pathology, inferior vena cava (IVC) anastomotic stenosis, and hepatic venous thrombosis, it is utilized in the setting of orthotopic liver transplantation (OLT) in adults [4]. TEE is more practical for intraoperative use than transthoracic echocardiography (TTE), as it avoids the surgical field and allows for preload assessment and real-time evaluation of cardiac contractility. The standard use of TEE in OLT has been advocated for in high volume liver transplant centers [5] but is often utilized based on the extent of the surgical procedure, the patient's medical comorbidities, and anticipated perioperative hemodynamic perturbations. Although the use of TEE as a monitoring tool is often utilized in patients with unanticipated hemodynamic instability, the use of TEE in pediatric patients for hepatic tumor resection has not been well described. Written HIPAA authorization has been obtained from the parent(s) or guardian(s) for this case report.

## 2. Case Description

**Figure 1:**
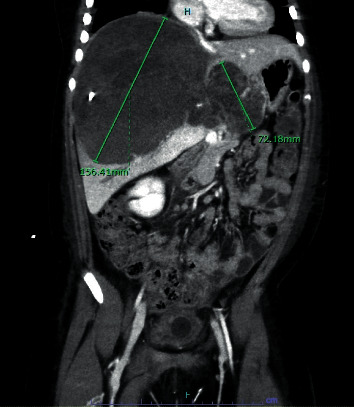
Coronal contrast-enhanced abdominal CT demonstrating large hepatic tumor with in situ drain in the right hepatic lobe.

**Figure 2:**
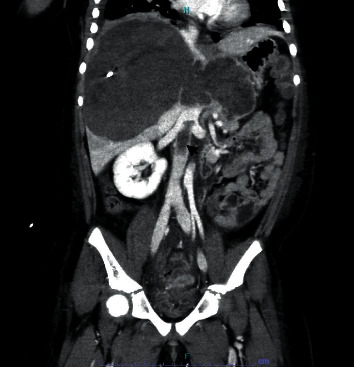
Coronal contrast-enhanced abdominal CT demonstrating IVC thrombus originating adjacent to the right renal hilum.

We discuss the case of a 44.5 kg, 8-year-old female with no significant past medical history who presented with abdominal pain and distension accompanied by nausea and vomiting. An ultrasound revealed a mixed solid and cystic 17 × 9 cm mass in the right liver lobe with compression of the IVC (see Figure 1). In light of worsening abdominal distention with concern for abdominal compartment syndrome, biopsy and drain placement of the mass was performed on hospital day 7. Over the next several days, persistent sanguineous drain output and resultant anemia were concerning for hemorrhage. CT revealed an infrarenal IVC thrombus (see Figure 2) and near-occlusive embolus distal to the left pulmonary artery. TTE was obtained revealing compression of the distal IVC at the right atrial junction with normal biventricular function and no evidence of elevated right heart pressures. Although heparin was initiated, there was significant difficulty in maintaining therapeutic anticoagulation in light of the underlying hemorrhagic liver mass as well as coagulopathy in the setting of synthetic liver dysfunction.

On hospital day 15, the patient underwent right hepatectomy with caudate lobe resection. In light of the patient's coagulopathy, IVC thrombus, pulmonary embolus (PE), and the anticipated use of intraoperative TEE, a pediatric cardiac anesthesiologist was requested. In addition, extracorporeal membrane oxygenation, a perfusionist, and the cardiac surgical team were also on standby. Blood products were placed on hold, and a rapid transfusion device was readied in the OR. The patient arrived to the OR with a double lumen PICC and two 20-gauge PIVs in situ. After preoxygenation, rapid sequence induction of anesthesia was performed with propofol 100 mg, ketamine 20 mg, fentanyl 100mcg, and rocuronium 60 mg. She was intubated without difficulty using a Mac 2 laryngoscope and 5.5 endotracheal tube. Anesthesia was maintained with sevoflurane at 1.4–1.8% with intermittent opioid boluses.

Subsequently, further invasive access was achieved with 6 F left internal jugular vein central line, 2.5 F left radial arterial line, and 16-gauge peripheral IV. In addition to standard ASA monitors, invasive arterial blood pressure and central venous pressure were monitored throughout the case. A TEE probe was utilized to assess the patient's cardiac function intermittently. These invasive monitoring modalities were chosen in light of the risk for significant rapid blood loss as well as potential embolization of the IVC thrombus and propagation of her existing PE.

**Figure 3:**
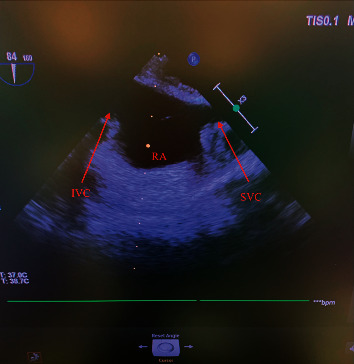
TEE bicaval view demonstrating a patent IVC without thrombus.

Surgical preparation was performed in a manner leaving her neck, chest, abdomen, and pelvis exposed for emergent intervention if necessary. Of note, her initial CVP was elevated to 17-18 mmHg but improved significantly to 1–3 mmHg after abdominal incision was made, likely due to the large size of the abdominal tumor and relief of pressure from abdominal ascites. In order to maintain oxygenation in light of the patient's PE, she was maintained on FiO_2_ of 1.0 throughout the case. Intraoperative TEE was utilized to monitor for right ventricle (RV) dysfunction and propagation or dislodgement of the IVC thrombus. In addition, the RV pressure gradient (PG) across the tricuspid valve was monitored to evaluate potential RV strain from a subsequent PE. At the initial exam, no thrombus was visualized in the RA and IVC on midesophageal (ME) bicaval view (see Figure 3).

Her intraoperative course was uncomplicated, and the patient remained hemodynamically stable throughout. Rotational thromboelastometry was sent off intraoperatively, and the patient was transfused 2 units of red blood cells and 1 unit of fresh frozen plasma. She also received 250 mL 5% albumin due to large volume ascites that was evacuated upon entrance into the abdomen. As the incision was being closed, a dexmedetomidine infusion was initiated at 1 mcg/kg/min. The patient was brought to the intensive care unit intubated and sedated on dexmedetomidine at 0.5 mcg/kg/min. She was extubated later that evening without complication. The patient was discharged on hospital day 28, with plans for initiation of a chemotherapeutic regimen.

## 3. Discussion

The differential diagnosis of UESL includes hepatic mesenchymal hamartoma, embryonal rhabdomyosarcoma of the biliary tree, and hepatoblastoma [6]. Following surgical resection, patients with negative resection margins and those receiving adjuvant chemotherapy showed improved overall and recurrence-free survival rates. Patients with unresectable tumors may benefit from neoadjuvant chemotherapy followed by tumor resection, or OLT in patients refractory to chemotherapy. Postoperative radiation therapy has been utilized in patients to prevent tumor bed recurrence, as well as for treatment of pulmonary and paraspinal metastases [6].

A number of case reports have discussed the utilization of TEE for vena cava resection and hepatectomy in adult patients [7]. Alcaraz et al. [7] visualized the intrahepatic IVC during a right hepatectomy with a modified bicaval view to diagnose Budd–Chiari syndrome intraoperatively. Standard perioperative TEE views (ME bicaval view and the transgastric view) can be utilized to evaluate intraoperative hemodynamic instability and diagnose anatomic or physiologic abnormalities during tumor resection. TEE can be utilized as a reliable monitor for intraoperative thromboembolic, air and fat emboli, presenting as significant hypoxia and hemodynamic instability [8].

Intraoperative TEE affords direct visualization of emboli in the pulmonary arteries and its proximal divisions. TEE findings suggestive of PE include RV hypokinesis, dilatation, tricuspid regurgitation (TR), abnormal motion of the interventricular septum, and an enlarged plethoric IVC [9]. In a large acute PE, RV hypokinesis is present in 92% of patients [10]. This is often accompanied by reduced left ventricle (LV) size and systolic function, reduced cardiac output, abnormal diastolic function, and a prominent A-wave on pulse wave Doppler recording of the mitral valve inflow [11].

When distal emboli are unable to be visualized, TEE can reveal acute RV overload, including McConnell's sign [12]. McConnell's sign is used to differentiate patients with acute PE from pulmonary hypertension due to other etiologies. It is caused by tethering of the RV apex to a hypercontractile, hyperdynamic LV apex. Patients present with an acute increase in afterload due to segmental ischemia of the RV free wall [13]. In this instance, RV enlargement and dysfunction were present with preservation of apical contractility.

An acute PE is a potentially fatal condition caused by partial or complete pulmonary artery obstruction from thrombus, tumor, fat or air, most commonly from lower extremity thrombi. Mechanical obstruction of pulmonary blood flow leads to elevated pulmonary vascular resistance (PVR) as well as platelet activation with release of both bronchoconstrictive and vasoconstrictive agents [14]. Although ventilation-perfusion mismatch with resultant hypoxemia is a common finding, mortality is related to acute hemodynamic compromise from elevated PVR and subsequent RV dysfunction. Early diagnosis and aggressive management reduces mortality to 3 to 8% [14].

**Figure 4:**
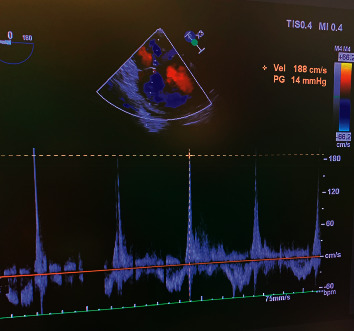
Doppler across the tricuspid valve demonstrating a peak gradient of 14 mmHg.

During this case, a technique described by Yock et al. [15] was utilized to estimate RV systolic pressure by monitoring for TR with Doppler ultrasound. Given the patient's IVC thrombus, there was a potential for significant embolic phenomena. Use of TEE enabled real-time monitoring of changes in RA and RV volume and function as well as visualization of clot propagation. Following liver mass resection, final images of IVC and RA patency confirmed that the RV size remained at baseline. In addition, the pulmonary artery systolic pressure was extrapolated through the TR jet measurement, which remained stable (see Figure 4). We concluded that if significant embolization occurred, PVR would be elevated leading to increased RV systolic pressure. As such, little to no increase in RV pressures was evident.

Continuous TEE surveillance can alert the clinician to embolization of an IVC tumor prior to the development of hemodynamic instability. In this challenging pediatric patient, the use of TEE was integral to guide management and diagnosis of a potential intraoperative PE.
